# Application of the Kushida morphometric model in patients with sleep-disordered breathing

**DOI:** 10.1016/S1808-8694(15)31002-8

**Published:** 2015-10-19

**Authors:** Maria Claudia Mattos Soares, Lia Rita de Azeredo Bittencourt, Adriane Iurck Zonato, Luis Carlos Gregório

**Affiliations:** a Otorhinolaryngologist and sleep medicine specialist, Master's degree graduate student at UNIFESP, collaborating physician of the otorhinolaryngology unit at FMABC; b Doctor in Medicine, Pneumologist and sleep medicine specialist; c Doctor in Medicine, Otorhinolaryngologist and sleep medicine specialist; d Doctor in Medicine, Head of the Otorhinolaryngology and Head & Neck Surgery Unit - UNIFESP. Sao Paulo Federal University

**Keywords:** obstrutive sleep apnea, mouth, measurements, obesity

## Abstract

The morphometric model is a useful screening test to investigate the possibility of OSAS in patients during initial office visits.

**Aim:**

To evaluate the clinical applicability of the Kushida morphometric model in a sample of patients with sleep-disordered breathing, and to define a cutoff value to differentiate patients with mild, moderate and severe apnea.

**Method:**

A sample of 80 patients with sleep respisratory disorder was studied. Patients were aged between 18 and 75 years, of both genders and had been submitted previously to polysomnography. The model cutoff value to distinguish between patients with or without apnea is 70.

**Results:**

In this sample, the model cutoff value in all four groups was less than 70. It was impossible to establish a cutoff value according to the gravity of the condition, due to the proximity and the nonlinear increase in the values presented by the nonapneic group and those with mild and moderate apnea.

**Conclusion:**

The Kushida morphometric model can be applied in clinical practice to a selected sample and it was impossible to establish a cutoff value to separate patients with obstructive sleep apnea-hypopnea syndrome according to severity.

## INTRODUCTION

Studies of the main characteristics of patients with Sleep Apnea-Hypopnea Syndrome (SAHS) started with Burwell, Robin, Waley and Bickelmann (1956)^1^. They described the Pickwickian Syndrome to honor the English novelist Charles Dickens, author of the famous “The Posthumous Papers of the Pickwickian Club” (1837), the main character of which was an obese, sleepy and snoring boy. The syndrome is classically composed of obesity, hypercapnia, cor pulmonale, erythrocytosis and excessive daytime sleepiness.

According to the definition published by the American Academy of Sleep Medicine (AASM) during a task force launched in 1999, SAHS is characterized by recurring episodes of partial or total obstruction of upper airways during sleep. It presents as a reduction (hypopnea) or cessation (apnea) of air flow, regardless of inspiratory effort, lasting at least 10 seconds. Hypopnea is defined as a more than 50% reduction in respiratory amplitude, or less than 50% if associated with oxygen desaturation over 3% and/or awakening (AASM Task Force, 1999)^2^.

In an important epidemiological study, Young, Palta, Dempsey, Skatrud, Weber and Badr (1993)^3^ concluded that SAHS has an increased prevalence in the obese, affecting 2% of women and 4% of men if we consider an apnea-hypopnea index (AHI) over 5 on polysomnography (PSG) and the presence of excessive daytime sleepiness; this condition is more frequent in middle-aged persons (Olson, King, Hensley and Saunders, 1995)^4^.

The pathophysiology of SAHS is still not fully understood; it is clear that the main phenomenon of the disease is pharyngeal collapse resulting in part from upper airway and facial skeletal anatomical alterations and reduced neuromuscular tonus (Arens and Marcus, 2004)^5^.

The main SAHS-related upper airway anatomical alterations are: tonsillar hypertrophy, a large tongue, medial tonsillary pillars, a posterior and/or thickened and/or a web (a membrane formed by the low insertion of the tonsillar pillar posterior to the uvula) soft palate, and a long and/or thickened uvula^6^, ^7^, ^8^, ^9^.

Many studies have been done assessing craniofacial dimorphism in SAHS patients using cephalometrics, computed tomography, magnetic resonance imaging, and acoustic reflection. Although there is controversy in the results, craniofacial alterations most closely related to the occurrence and severity of SAHS are: retroposition of the maxilla, shortening of the mandibular body, inferiorly displaced hyoid bone, retrognatism, dental occlusion class II (mesial sulcus of the 1st permanent lower molar articulates posteriorly with the mesiovestibular cusp of the 1st permanent upper molar) and ogival hard palate^5^, ^7^, ^8^, ^9^, ^10^, ^11^, ^12^.

Currently SAHS is considered a chronic, progressive and incapacitating disease with high morbidity and mortality, since it is associated with an increased risk of cardiovascular diseases, traffic accidents and depression. It is considered a public health issue^13^, ^14^, ^15^, ^16^.

PSG is an essential assessment and diagnosis tool in Sleep Medicine; however, it is a time-consuming and expensive exam requiring expert technicians, which frequently limits its use, with the result that SAHS is underdiagnosed. Young, Evans, Finn and Palta (1997)^17^ estimated that 93% of women and 82% of men with moderate or severe SAHS are not diagnosed by physicians.

Various investigators have developed predictive clinical rules for SAHS using mathematical formulae including variables such as the body mass index (BMI), the cervical circumference (CC), oxygen saturation, the AHI and questionnaires^6^, ^7^, ^18^, ^19^, ^20^, ^21^. However, these models do not take into account craniofacial alterations, and some authors consider them insufficient^22^. Kushida, Efron and Guilleminault (1997)^22^ proposed a morphometric model (MM) described below, which includes measurements of the mouth, the BMI and the CC to predict which patients would be at an increased risk for SAHS (and therefore would require PSG promptly). This model was applied to 300 patients (224 men and 76 women) aged between 15 and 75 years. All of them had undertaken PSG, but the examiner did not have access to the results of this exam. The criteria for diagnosing SAHS was the presence of typical symptoms (snoring, respiratory pauses, excessive daytime sleepiness, Epworth's Sleepiness Scale > 10, and an AHI > 5.

{P + (Mx - Mn) + 3 x OJ} + 3 x [Max (IMC - 25)] x (CC / IMC)

P: palatal height in millimeters

Mx: distance between the mesial side of the upper 2nd molars in millimeters

Mn: distance between the mesial side of the lower inferior 2nd molars in millimeters

OJ: overjet (horizontal distance between the incisor side of the upper and lower central incisors)

BMI: weight (kilograms)/height (meter2)

CC: cervical circumference in centimeters

In patients with a BMI ≤ 25 only the first part of the formula was applied (between wavy brackets) in such a way that the second part of the formula (between brackets) was always zero or a positive number.

Following application of the MM, patients were divided into two groups: one without SAHS (MM< 70) and one with SAHS (MM≥ 70); 46 patients were allocated to the first group and 254 to the second group. The MM classified six patients with SAHS as non-apneic. Sensitivity was 97%, specificity was 100%, the positive predictive value was 100%, and the negative predictive value was 88.5%.

The BMI and the CC were also used to divide the patients into both groups, with cutoff points of 25 kg/m2 (BMI) and 40cm (CC). We compared the individual capability of the MM, the BMI and the CC to identify patients with and without SAHS; the MM was superior, followed by the BMI and the CC.

The authors conclude that the MM is a quick and safe form of screening patients that will require specialized investigation such as PSG to establish the severity of the disease and to guide the appropriate treatment.

The aims of this study were to assess the clinical applicability of the Kushida MM in a sample of patients with sleep-related respiratory disorders (SRDs) and to define a cutoff point to identify patients with mild, moderate and severe apnea.

## METHODS

We studied 80 patients from the Sleep Disorders Outpatient Clinic of the Otorhinolaryngology (ORL) and Head & Neck Surgery (HNS) Unit at the Sleep Institute of the Sao Paulo Federal University (UNIFESP) between September 2003 and August 2004. All patients underwent prior polysomnography and accepted the free and informed consent form proposed. The study was approved by the UNIFESP-EPM Research Ethics Committee.

### 1 Inclusion criteria

• patients complaining of snoring, apnea and/or excessive daytime sleepiness
• adults aged between 18 and 75 years
• both genders
• patients having undertaken a prior polysomnography.

### 2 Exclusion criteria

• a history of benign or malignant upper airway neoplasms
• patients with limited mouth opening
• absence of upper and/or lower central incisors
• absence of the upper and lower 2nd molars
• patients unable or unwilling to cooperate with the study
• prior treatment for SAHS
• the use of illegal drugs, alcohol and hypnotic medication.

### 3 Selection of patients

The 80 patients were selected from the Sleep Disorders Outpatient Clinic of the ORL and HNS Unit at UNIFESP during consultation with an otorhinolaryngologist or when undergoing PSG. There were 20 patients with mild SAHS (5>AHI≤15), 20 patients with moderate SAHS (15>AHI≤30), 20 patients with severe SAHS (AHI >30 and 20 patients with a normal AHI (AHI≤5).

### 3 Polysomnography

Polysomnography was an overnight recording of the patients sleep with monitoring by an electroencephalogram (EEG), an electrooculogram (EOG), a tibialis and mentalis electromyogram (EMG), an electrocardiogram (ECG), measurement of airflow through a nasal cannula and an oral thermistor, breathing movements measured by thoracoabdominal belt, oxymetry for SaO2, and microphone recordings of snoring. We used the SAC-Oxford, version 10.0 polysomnograph.

EEG, EOG and EMG interpretation defined sleep architecture stages according to the criteria proposed by Rechtschtaffen and Kales (1968).

The staging of respiratory events followed the criteria proposed by the AASM (AASM Task Force, 1999).

### 5 Assessment of patients

After selection, age, gender, weight, height, and frequency of snoring (nightly, nearly every night, occasionally or never) were recorded. Patients with an AHI ≤ 5, but which reported snoring every night or nearly every night, were classified as snorers. Patients that snored occasionally or never were classified as non-snorers if PSG recorded intermittent snoring; if PSG recorded constant snoring, these patients were classified as snorers.

Next, Kushida's MM was applied. Patients were placed in the Frankfurt position and standard tools were used; all measurements were made by the same examiner. Measurements of the mouth, except for overjet (OJ), were made with the tongue in a relaxed position and the mouth open at a 20^o^ angle with the mandibular condyle. A compass with a fixed 20^o^ angle was placed over the temporomandibular joint; the tip of the upper leg was aligned with the upper central incisors and the tip of the lower leg was aligned with the lower central incisors, thus obtaining the desired mouth opening. A second compass was used to obtain the mouth measurements (the maxilla intermolar distance - Mx, the mandible intermolar distance - Mn and the palatal height - P), which were then transposed to a ruler. A 40 millimeter (mm) ruler was used to measure the OJ, and a tape measure was placed over the cricothyroid membrane to measure the CC.

### 6 Forms of assessing the results

Variables were expressed as the average, the standard deviation, and minimum and maximum values, which showed a normal distribution.

ANOVA was used for comparison between groups; Ducan's post-test was used if needed to separate the groups.

The Student's T test for independent samples was used for comparisons between two groups.

Peason's test was used to assess correlations between variables.

The multivariate regression analysis was used to assess factors that could define the weighting of some measurements.

## RESULTS

Ages and the BMI of the 80 patients are shown on Table 1. There were more men than women (54 men/26 women); the average age was 40.5 ± 13.6 years, and on average the patients were slightly overweight.

**Table 1:** Age and BMI of the 80 patients.

Variables	Age (years)	BMI (Kg/m2)
Average	40,5	27,4
Standard Deviation	13,6	4,9
Minimum Value	18,0	17,0
Maximum Value	71,0	45,0

BMI: body mass index

Data on orofacial and CC measurements and final MM values of the 80 patients are on Table 2.

**Table 2:** Orofacial and Cervical Circumference Measurements and Morphometric Model Values of the 80 patients.

Variables	P	Mx	Mn	OJ	CC	MM
Average	31,3	37,2	36,4	3,23	40,4	55,1
Standard Deviation	6,3	4,5	6,1	2,65	4,1	18,1
Minimum Value	20,0	27,0	4,0	0,0	31,0	26,0
Maximum Value	48,0	51,0	46,0	20,0	54,0	115,5

P: palatal height in millimeters

Mx: distance between the mesial side of the upper 2nd molars millimeters

Mn: distance between the mesial side of the lower 2nd molars millimeters

OJ: overjet in millimeters

CC: cervical circumference in centimeters

MM: morphometric model values

A comparison between the averages of all variables for the four groups in this study is shown on Table 3. The following variables were significant in the comparison of the four groups of patients (p ≤ 0.05): age, BMI, E1 and 2, SaO2 min, OJ, CC and MM. The following variables were not significant (p > 0.05): ES, E3 and 4, REM, P, Mx and Mn.

**Table 3:** Average and standard deviation of variables in subgroups.

Variables	Non-apneic	SD	Mild SAHS	SD	Moderate SAHS	SD	Severe SAHS	SD	p
	n = 20		n = 20		n = 20		n = 20		
Age (years)	35,2	13,3	37,9	15,9*	47,2	11,9•*	41,2	10,6	0,027
Gender (M/F)	5/15		15/5		18/2		16/4		
BMI	25,5	5,7□	25,5	2,7□	27,3	3,2*	31,4	5*□	0,0001
ES	81,5	11,3	80,7	12,8	82,6	14	83,2	9,9	0,920
E1 e 2	63,4	7,5□	62,9	10□	65,5	8,7□	78,3	9,2□	0,000001
E3 e 4	20,3	6,4	19,4	8,7	15,2	7,17	21,6	24,7	0,506
REM	16,7	6,6	17,9	5,9	19,7	6,2	16,7	6,8	0,132
SaO2 min	89,6	2,9□	86,3	5□	77,9	8,8	67,2	10,8□	0,000000
P	31,6	7,1	31	5,6	33,3	4,3	29,2	7,5	0,230
Mx	39,2	4,6	36,3	4,4	36,7	4,8	36,6	4	0,152
Mn	34,8	8,5	36,7	5	37,9	5,1	36,2	5,1•	0,444
OJ	2,9	1,3	3,7	2,1	2,61	1,8	3,6	4,4	0,41
CC	36,7	3,7*□	40,3	3,1*	41,2	2,2•□	43,6	3,9•*	0,000000
MM	52,4	14,5	50,3	17*	51,7	14,7•	66,7	22,3•*	0,013

Analysis of Variance ANOVA; Duncan's Post-Test

n: number of patients; P: palatal height in millimeters

DP: standard deviation Mx: distance between the mesial side of the upper 2nd molars millimeters

M: male; Mn: distance between the mesial side of the lower 2nd molars millimeters

F: female; OJ: overjet in millimeters

SAHS: obstructive sleep apnea-hypopnea syndrome; CC: cervical circumference in centimeter

BMI: body mass index in Kg/m2; MM: morphometric model values

ES: sleep efficiency as a percentage

E1 and 2: sum of NREM sleep stages 1 and 2 as a percentage

E3 and 4: sum of NREM sleep stages 3 and 4 as a percentage

• p < 0.05 REM: REM sleep as a percentage

* p < 0.01 SaO2 min: minimum oxyhemoglobin saturation as a percentage

□ p < 0.001 AHI: apnea-hypopnea index per hour of sleep

Variables were compared according to gender and whether snoring was present or absent; differences are shown on Tables 4 and 5. Variables that were statistically significant regarding gender were: AHI, SaO2 min, Mn and CC. Similar results regarding snoring were: age, BMI, SaO2 min, AHI and CC.

**Table 4:** Average of variables according to gender.

Variables	Gender				p
	Male (n = 54/67,5%)	SD	Female (n = 26/32,5%)	SD	
AHI	29,4	27,1	13,3	21,3	0,008 *
SaO2 mín	78,1	11,4	84,7	9,8	0,013 *
Mn	37,5	5,1	34,4	7,3	0,035 *
CC	41,9	2,6	37,5	4,8	0,000001*

n: number of patients

AHI: apnea-hypopnea index by hours of sleep

SaO2 min: minimum oxyhemoglobin saturation as a percentage

Mn: distance between the mesial side of lower 2nd molars in millimeters

CC: cervical circumference in centimeters

* Significant findings by the chi-squares test (p<0.05)

**Table 5:** Average of variables according to snoring.

Variables	Snoring				p
	Present (n = 74 /92,5%)	DP	Absent (n = 6 /7,5%)	DP	
Idade	41,4	13,6	28,3	6,1	0,02 *
IMC	27,8	4,7	21,8	1,9	0,003 *
SaO2 mín	79,5	11,3	90,8	3,9	0,017 *
IAH	25,7	26,6	2,7	4,1	0,038 *
CC	40,9	3,7	34,5	3,5	0,0001 *

n: number of patients

BMI: body mass index

SaO2 min: minimum oxyhemoglobin saturation as a percentage

AHI: apnea-hypopnea index by hours of sleep

CC: cervical circumference in centimeters

* Significant findings by the chi-squares test (p < 0.05)

**Figure 1:**
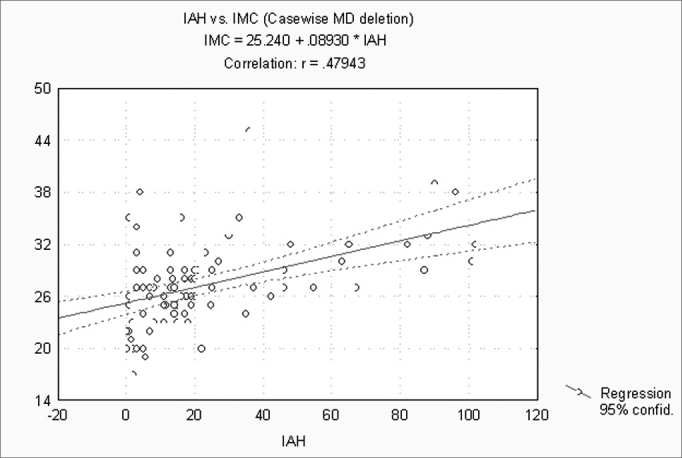
AHI x BMI - AHI: apnea-hypopnea index; BMI: body mass index

**Figure 2:**
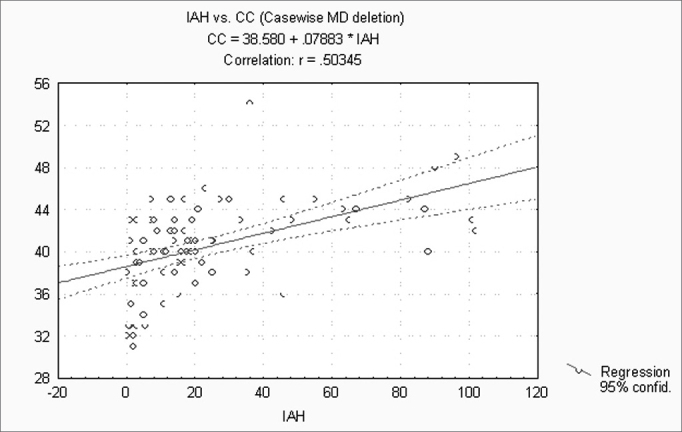
AHI x CC - AHI: apnea-hypopnea index; CC: cervical circumference

**Figure 3:**
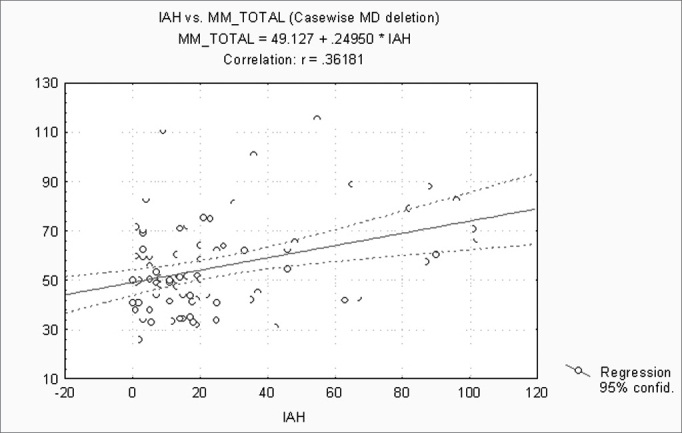
AHI x MM - AHI: apnea-hypopnea index; MM: morphometric model

**Figure 4:**
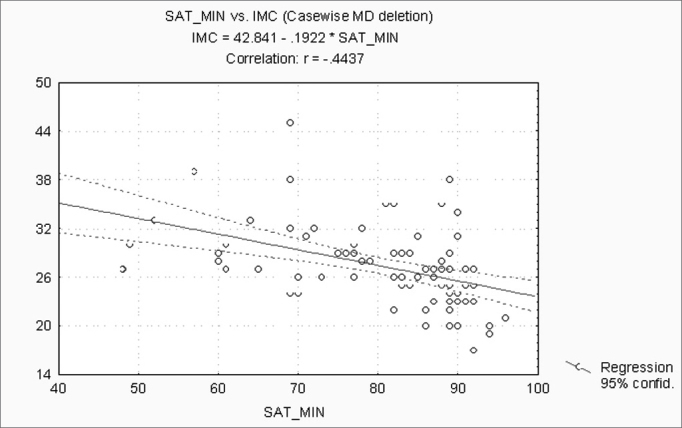
SaO2min x BMI - SaO2min: minimum oxyhemoglobin saturation; BMI: body mass index

**Figure 5:**
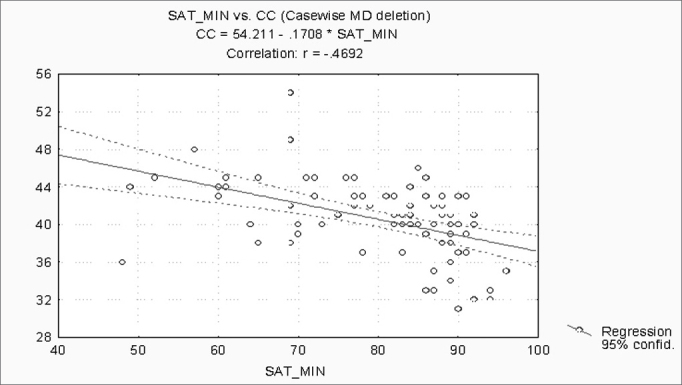
SaO2min x CC - SaO2min: minimum oxyhemoglobin saturation; CC: cervical circumference

**Figure 6:**
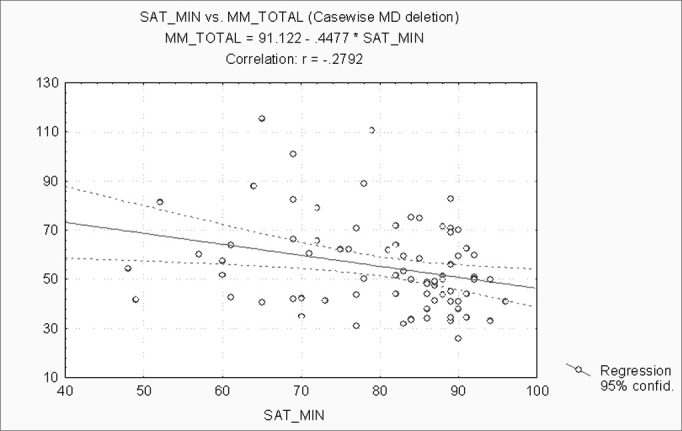
SaO2min x MM - SaO2min: minimum oxyhemoglobin saturation; MM: morphometric model

When correlating the AHI with the BMI, P, Mx, Mn, OJ, CC and MM, statistically significant variables (p ≤ 0.05) were: BMI, CC and MM (Figures 1, 2 and 3). Statistically significant variables were the same for SaO2 min (p ≤ 0.05) (Figures 4, 5 and 6).

The AHI, the SaO2 min, and other variables were analyzed by multivariate linear regression. The CC was significant among them to establish the AHI(p = 0.03). The Mx was significant (p = 0.016) to establish the SaO2 min.

## DISCUSSION

The average age of the patients was 40.5 ± 13.6 years, being higher in the moderate SAHS group. This is similar to reports in literature showing a higher prevalence of the disease between ages 40 and 60. There was a statistically significant difference between the ages of moderate SAHS patients compared to the non-apneic and mild SAHS groups, confirming the increased severity of the disease with age. This is a result of increased muscle flaccidity associated with decreased neuromuscular tonus, making the pharynx prone to collapse and apnea^3^, ^4^, ^22^.

There were more men than women in an approximately 2:1 proportion. Looking at the groups separately, there were more men only in the apnea groups; this is similar to published papers that reveal a greater prevalence of the disease in men^3^, ^4^.

The average BMI was 27.4 ± 4.9 kg/m2, characterizing the group as overweight (BMI between 25 and 29.9 kg/m2) according to the World Health Organization (1998). The severe SAHS group had statistically significant differences compared to the moderate and mild SAHS groups and the non-apneic group, demonstrating that the BMI is related to the occurrence and severity of the disease^3^, ^6^, ^8^.

There was a statistically significant difference between groups in the SaO2 min parameters measured during polysomnography. The pharynx in the SAHS tends to collapse more easily, leading to reduced air flow and a lower SaO2 min; thus, disease severity is proportional to oxyhemoglobin desaturation.

SAHS increases sleep stages 1 and 2, and reduces sleep stages 3 and 4 and REM sleep, as seen on Table 3. However there was a statistically significant difference between groups only in stages 1 and 2.

The percentage of slow-wave sleep falls gradually from age 20 onwards. This might explain why the moderate SAHS, which had a higher average age, had a lower percentage of stages 3 and 4.

There was no statistically significant difference in mouth measurements between our four groups. Mouth measurements (P, Mx and Mn) has lower average values compared to those described by Kushida, Efron and Guilleminault (1997)^22^. In their study, these authors found a statistically significant difference between patients with and without SAHS. These measurements are strongly related to genetic factors and/or to oral breathing. The populations under study in our groups and in Kushida, Efron and Guilleminault's study belong to different origins (Latin and North-Americans), there was no standardization to account for ethnicity, and some of our patients came from the Sleep Disorders Outpatient Clinic of the ORL Unit (which might have led to the selection of a higher number of oral breathers). These facts may explain the differences between both studies.

According to findings by Kushida, Efron and Guilleminault (1997)^22^, and Schellenberg, Maislin and Schwab (2000)^8^, there was a statistically significant difference in measurements of the overjet between subgroups in our sample.

The average cervical circumference was 40.4 ± 4.1 cm, and there was a statistically significant difference between the subgroups, with increasing values according to the severity of the obstructive respiratory sleep disorder, confirming published findings that correlate the CC with the occurrence and severity of SAHS^19^. Kushida, Efron and Guilleminault (1997)^22^ established a cutoff point of 40 cm in CC to separate patients with and without SAHS. In our study the average CC in non-apneic patients was 36.7cm (31 to 43cm) and the average CC for SAHS patients was over 40cm, a statistically significant difference coinciding with the values published by the abovementioned authors.

In our study the MM cutoff point was 70. The values we found were below 70, however the severe SAHS group came closest to this number (66.7 average), a statistically significant difference compared to the other groups. Although there was a significant difference between the subgroups, we were unable to establish a cutoff point according to disease severity, as the values were very similar and did not increase linearly from the non-apneic to the mild and moderate SAHS groups. On the other hand, the authors of the MM state in their paper that, similar to other predictive models, the MM is useful to screen and identify the more severe cases; these patients may then be prioritized for treatment. Furthermore, the authors also underline the importance of PSG as an adequate tool for diagnosis and eventual treatment.

Variables that showed differences when compared according to gender were the AHI, the SaO2 min, the CC and the Mn, all of them indicating increased severity in men. The male gender is an important risk factor for the occurrence and severity of SAHS^2^, ^3^, ^8^. Thus one would expect that disease-related variables such as the AHI, the SaO2 min and the CC would have statistically significant values. Furthermore, men tend to accumulate fat in the neck, and the CC provides a better correlation with the presence of SAHS than the body weight^19^. The only mouth measurement that was different between men and women was the Mn (37.5 ± 5.1 cm in men and 34.4 ± 7.3 cm in women). After the paper on which our study is based was published, and until our last bibliographical survey, there was no publication reproducing the MM, which restricts comparisons of our data. New studies are needed on this theme so that we may assess the correlation between the new proposed measurements (P, Mx and Mn) and obstructive respiratory sleep disorders.

92.5% of patients complained of snoring. Such a high prevalence may be explained by the fact that this is a selected population with obstructive respiratory sleep disorders. Comparing variables according to the presence or absence of snoring, we observed that age, BMI, SaO2 min, AHI and CC had statistically significant differences in both groups, with increased severity in patients with a complaint of snoring. This is expected, as emphasized above concerning the importance of each of these variables in SRD.

Separately correlating the AHI and the SaO2 min with the BMI, P, Mx, Mn, OJ, CC and the MM, we found that the statistically significant variables were the BMI, the CC and the MM, for both analyses. The AHI and the SaO2 min are strongly linked to SAHS. The variables that best correlate with obstructive respiratory sleep disorders according to medical literature are the BMI and the CC^7^, ^11^, ^18^, ^19^, ^21^. Our results confirm findings published by these articles.

Individually, measurements of the mouth (P, Mx, Mn and OJ) have no statistically significant correlation with the AHI and the SaO2 min; however, when grouped in the MM, this method shows a strong positive correlation. Calculations in the MM includes measurements of the mouth, the CC and the BMI, which may justify the results and underline the interaction between factors involved in the pathophysiology of SAHS.

Multivariate linear regression analysis revealed that the CC was significant to establish the AHI. This finding, together with the fact that the CC was the only measurement which increased proportionally to the severity of the disease (with a statistically significant difference between all groups), and which correlated with the AHI and the SaO2 min, supports the importance of the relationship between the CC and SAHS, as described previously.

A similar analysis using the SaO2 min showed that the Mx is the most significant measurement. The paucity of studies on direct measurements of the facial skeleton hinders comparisons with findings in literature, but underlines the value of craniofacial dimorphism in SAHS, as shown in numerous papers^5^, ^7^, ^8^, ^9^, ^10^, ^11^, ^12^.

## CONCLUSION

1. The morphometric model proposed by Kushida, Efron and Guilleminault is applicable to the selected sample in clinical practice.
2. It was not possible to establish a cutoff point to classify patients with sleep apnea-hypopnea syndrome according to severity.
